# Physiological Determinants of High Nitrogen-Use Efficiency in Waxy Sorghum: Integrated Analysis of Biomass Production, Nitrogen Assimilation, and Antioxidant Metabolism

**DOI:** 10.3390/plants15193037

**Published:** 2026-10-04

**Authors:** Jie Gao, Qingfeng Li, Can Wang, Qiang Zhao, Fangli Peng, Siyu Chen, Lingbo Zhou, Guobing Zhang, Mingbo Shao

**Affiliations:** Institute of Upland Food Crops, Guizhou Academy of Agricultural Sciences, Guiyang 550006, China; gaojie396300520@163.com (J.G.); wangc.1989@163.com (C.W.); 15761633106@163.com (Q.Z.); pfl9367@126.com (F.P.);

**Keywords:** waxy sorghum, nitrogen-use efficiency, physiological traits, leaf area index, yield

## Abstract

Sorghum [*Sorghum bicolor* (L.) Moench] production faces persistent challenges from low nitrogen-use efficiency (NUE) under the intensive cultivation systems of Southwest China. To elucidate the physiological determinants of genotypic variation in NUE, a two-site field experiment was conducted in Guiyang and Anshun using a high-NUE waxy sorghum cultivar (HLF) and a low-NUE waxy sorghum cultivar (QV9) under two nitrogen levels: no nitrogen application (BSN, 0 kg N ha^−1^) and conventional nitrogen application (SN, 150 kg N ha^−1^). Results showed that grain yield (Yield) and NUtE responded significantly to genotype (G), nitrogen application rate (N), and G × N effects across both sites. For NUpE, the main effect of N was significant at Anshun but not at Guiyang, whereas genotypic differences were consistent across both sites. Under BSN treatment, HLF exhibited a modest 11.04% yield reduction relative to SN, whereas QV9 suffered a 24.31% decline. Across both nitrogen levels and sites, HLF consistently outperformed QV9 in NUpE and NUtE, with NUpE reaching 1.58–1.98 times that of QV9. Nitrogen application significantly enhanced leaf area index (LAI) and SPAD values, and HLF maintained higher canopy indices at post-anthesis compared to QV9. Total dry matter and nitrogen accumulation (TDMA and TNA), post-anthesis dry matter and nitrogen accumulation (DMAP and NAP), and proportion of post-anthesis dry matter and nitrogen accumulation (DMAPP and NAPP) of HLF were higher than QV9. Although the BSN-HLF produced higher dry matter translocation amounts than BSN-QV9, HLF exhibited lower dry matter and nitrogen translocation rates (DMTR and NTR) and contribution rates of dry matter and nitrogen to grains (DMTCR and NTCR). At the enzymatic level, HLF sustained significantly elevated activities of nitrate reductase (NR), glutamine synthetase (GS), and glutamate synthase (GOGAT) and maintained elevated antioxidant enzyme activities (SOD and POD) at both anthesis and maturity stages under BSN treatment. Concurrently, HLF accumulated higher concentrations of osmolytes (proline and soluble sugars), while maintaining reduced malondialdehyde (MDA) content, indicative of strengthened cellular homeostasis under BSN treatment. Mantel tests further revealed that DMAPP, GS, GOGAT, POD, SSC, and MDA were commonly and significantly linked to both NUE and yield. Overall, HLF maintained higher NUE and yield stability under low nitrogen, which may be associated with its sustained nitrogen metabolic enzyme activities, optimized canopy performance, and enhanced antioxidant and osmotic adjustment capacities.

## 1. Introduction

Nitrogen, a fundamental macronutrient essential for plant growth and development, serves as a critical constituent of key biomolecules including proteins, nucleic acids, and chlorophyll. It plays a decisive role in regulating photosynthesis, biomass accumulation, and ultimately yield formation [1,2]. Globally, agricultural production remains heavily reliant on synthetic N fertilization to ensure food security and meet the demands of a growing population [3,4]. However, the low recovery efficiency of applied N fertilizer—typically ranging from 30% to 50% [5,6]—results in substantial losses via leaching, volatilization, and denitrification. Such inefficiencies not only represent a significant waste of resources and increased economic costs but also trigger severe ecological consequences, including water eutrophication, soil acidification, and greenhouse gas emissions [7,8]. Consequently, elucidating the intrinsic mechanisms underlying high nitrogen-use efficiency (NUE) in crops, coupled with breeding N-efficient cultivars and optimizing N management strategies, constitutes a pivotal pathway toward sustainable agriculture and global food security [9,10].

As a premier C4 cereal, sorghum [*Sorghum bicolor* (L.) Moench] combines remarkable resilience to drought and nutrient-poor soils with superior photosynthetic capacity, making it indispensable for agriculture on marginal lands and in arid zones globally [11,12,13]. Its utility extends beyond food security, serving critical roles in forage and bioenergy sectors [14]. Against the backdrop of intensifying climate change, sorghum’s strategic value is poised to increase. Regionally, Guizhou Province represents a unique case study. As the epicenter of Chinese Baijiu production, the province cultivates predominantly waxy sorghum across a stable area of ~13.33 × 10^4^ hectares [15,16]. Yet, this industrial boom has precipitated a reliance on conventional high-input farming systems characterized by excessive N fertilization. Alarmingly, while extensive studies exist for rice, wheat, and maize, the genetic variability in NUE among waxy sorghum genotypes remains largely uncharacterized, especially under the specific ecological conditions of Southwest China. This knowledge gap impedes efforts to optimize agronomic management and genetic improvement. Therefore, dissecting the unique physiological architecture of nitrogen uptake, assimilation, translocation, and remobilization in Guizhou waxy sorghum is of paramount importance. This endeavor promises to unveil the mechanistic basis of N efficiency in waxy sorghum, thereby guiding precision cultivation and accelerating molecular breeding programs. Ultimately, these insights will bolster the economic viability of specialty grain industries across Southwest China’s mountainous terrains.

Genetic improvement of crop nitrogen-use efficiency (NUE) relies on assessing nitrogen uptake, assimilation, allocation, and translocation [17,18]. Plant nitrogen status and photosynthetic carbon assimilation are tightly and bidirectionally regulated, and leaf-age-dependent nitrogen allocation strongly affects canopy light use [19,20,21]. In rice and other crops, high-NUE genotypes usually exhibit higher post-anthesis nitrogen accumulation and more efficient transfer of nitrogen from stems, sheaths, and leaves to grains [22,23]. Activities of rate-limiting enzymes such as nitrate reductase (NR) and glutamine synthetase (GS) are associated with genotypic variation in N metabolism [24,25]. Under low-N conditions, sorghum can maintain C–N metabolic homeostasis by adjusting root architecture, photosynthetic traits, and N-metabolic enzyme activities (NR, NiR, GS, GOGAT) [26]. For quantitative evaluation, the NUE decomposition framework of Moll et al. [27] and the incremental efficiency concepts of Cassman et al. [28] provide a theoretical basis for assessing crop N efficiency, and N-efficient genotypes generally show greater yield advantage under low N.

Despite extensive insights into these physiological processes in model species and major cereals, their specific manifestation in waxy sorghum remains largely uncharacterized. Critical uncertainties remain concerning the responsiveness of carbon assimilation to N availability, the precise kinetics of N remobilization across organs, and the temporal regulation of key enzymatic activities. Crucially, the integrative physiology linking these processes to NUE metrics requires further investigation. To address this, we conducted a comparative analysis using waxy sorghum genotypes with divergent NUE phenotypes under contrasting nitrogen applications. We examined the associations between biomass production, N dynamics, the enzymatic machinery of N metabolism, and protective mechanisms (including antioxidant enzymes and osmolyte accumulation). This integrated analysis aims to identify the key physiological traits associated with N efficiency and their coordinated patterns across growth stages. Ultimately, this work provides physiological insights essential for optimizing agronomic management and accelerating molecular breeding programs targeting enhanced NUE in this strategically important crop.

## 2. Results

### 2.1. Grain Yield and Its Components

Two-way ANOVA revealed that grain yield was significantly affected by genotype, nitrogen application rate, and their interaction (*p* < 0.05) (Table 1). Under the BSN treatment, the grain yield of HLF reached 5861.34 kg·ha^−1^ (6094.13 kg·ha^−1^ at Anshun), representing an 11.04% (10.48% at Anshun) reduction compared to the SN treatment. In contrast, QV9 yielded 2992.95 kg·ha^−1^ (2757.23 kg·ha^−1^ at Anshun) under BSN, showing a sharper decline of 24.31% (26.31% at Anshun) relative to SN. Yield components were also influenced by both genotype and nitrogen application rate. HLF consistently outperformed QV9 across all parameters. Under BSN, reductions in yield components were observed at the Anshun site: HLF exhibited 2806.77 grains per panicle, a grain weight per panicle of 53.19 g, and a 1000-grain weight of 18.95 g, whereas the corresponding values for QV9 were markedly lower (1559.20, 26.83 g, and 17.21 g, respectively).

### 2.2. Nitrogen-Use Efficiency (NUE)

As shown in Table 2, the N uptake efficiency (NUpE) of HLF was significantly higher than that of QV9 under both SN and BSN treatments (*p* < 0.05), ranging from 0.41 to 0.87 kg·kg^−1^. Under the SN treatment, the NUpE of HLF reached 0.71 kg·kg^−1^ (Guiyang) and 0.80 kg·kg^−1^ (Anshun), which were 1.58-fold and 1.78-fold higher than those of QV9, respectively. Under BSN, this advantage increased further, with HLF exhibiting 1.80-fold and 1.98-fold higher values than QV9 at the respective sites. Significant genotypic differences in NUtE were also observed under BSN treatment, where HLF increased by 7.29% (Guiyang) and 11.15% (Anshun) compared to QV9. Regarding agronomic indices, the nitrogen partial factor productivity (NPFP) and nitrogen recovery efficiency (NRE) of HLF under SN treatment were 43.92 kg·kg^−1^ and 66.36% (Guiyang) and 45.39 kg·kg^−1^ and 69.65% (Anshun), respectively. These values were significantly higher than those of QV9 (26.36 kg·kg^−1^ and 50.62% in Guiyang; 24.94 kg·kg^−1^ and 47.43% in Anshun).

### 2.3. Leaf Area Index (LAI) and Chlorophyll Content (SPAD)

As shown in Figure 1, both LAI and SPAD values exhibited a unimodal curve across the growth stages, peaking at anthesis. Nitrogen application significantly increased the LAI and SPAD at all growth stages across both sites (*p* < 0.01). Notably, HLF maintained a higher LAI at the jointing stage than DAA30 compared to QV9, and exhibited a slower post-anthesis decline in LAI under the BSN treatment. At the DAA30 stage, LAI of HLF remained at 3.30 (Guiyang) and 3.96 (Anshun), representing an average reduction of only 21.54% from the anthesis peak, whereas QV9 showed a steeper decline (27.24%). Consistent with the LAI trend, SPAD values were persistently higher in HLF throughout the growth period under BSN treatment.

### 2.4. Dry Matter Accumulation and Translocation

As shown in Table 3, the total dry matter accumulation (TDMA), post-anthesis dry matter accumulation (DMAP), and grain dry matter accumulation (GDMA) were significantly affected by nitrogen application rate and genotype at both experimental sites. Overall, these parameters followed the order: SN > BSN and HLF > QV9. Notably, the BSN−HLF resulted in significantly higher values for these three indices compared to the SN−QV9, with average increases of 30.25%, 15.72%, and 28.71%, respectively. While no significant genotypic difference in the proportion of post-anthesis dry matter accumulation (DMAPP) was observed under SN at the Anshun site, HLF exhibited a significantly higher DMAPP than QV9 under BSN, increasing by 10.47 percentage points. The harvest index (HI) showed no significant variation among treatments at Guiyang but differed significantly at Anshun, following the order: SN−HLF > SN−QV9 ≈ BSN−HLF > BSN−QV9.

Figure 2 illustrated that the dry matter translocation amount (DMTA), translocation rate (DMTR), and contribution rate to grains (DMTCR) were significantly higher under BSN than under SN, with this trend being particularly pronounced at the Anshun site. Under BSN, these parameters in HLF reached 2.91, 3.33, and 4.32 fold of their respective SN values, while those in QV9 reached 2.83, 3.31, and 5.05 fold. Interestingly, the DMTR and DMTCR of HLF were consistently lower than those of QV9 regardless of nitrogen treatment. This suggests that QV9 relied more heavily on pre-anthesis assimilate translocation to support grain filling compared to the nitrogen-efficient HLF.

### 2.5. Nitrogen Accumulation and Translocation

As shown in Table 4, total nitrogen accumulation (TNA), post-anthesis nitrogen accumulation (NAP), grain nitrogen accumulation (GNA), and proportion of post-anthesis nitrogen accumulation (NAPP) were significantly affected by genotype and nitrogen application rate (*p* < 0.05). Regardless of nitrogen treatment, HLF exhibited significantly higher values for all parameters compared to QV9. This advantage was particularly evident for post-anthesis nitrogen accumulation and its proportion. Under BSN, HLF significantly outperformed SN-QV9, with post-anthesis nitrogen accumulation increasing by 50.05% (Guiyang) and 41.60% (Anshun), and the proportion of post-anthesis nitrogen accumulation (NAPP) increasing by 10.82 and 5.94 percentage points, respectively. The nitrogen harvest index (NHI) also differed significantly between genotypes; under BSN, HLF showed increases of 11.7% (Guiyang) and 32.93% (Anshun) compared to QV9, while under SN, the increases were 13.42% and 10.77%, respectively.

Figure 3 illustrated the genotypic differences in nitrogen translocation characteristics. While no significant differences were observed in nitrogen translocation amount (NTA) among treatments, the nitrogen translocation rate (NTR) and its contribution to grains (NTCR) varied significantly. QV9 consistently exhibited higher NTR and NTCR than HLF under both SN and BSN treatments. Specifically, under BSN treatments, QV9 reached 28.74% and 57.63% for NTR and NTCR at the Guiyang site, and 21.30% and 52.16% at the Anshun site, respectively. These results indicate that, in the absence of significant differences in nitrogen translocation amounts, the nitrogen-inefficient genotype QV9 relied more heavily on nitrogen remobilization from vegetative organs.

### 2.6. Activities of Nitrogen Metabolism Enzymes

The genotypic differences in nitrogen metabolism enzyme activities corroborated the observed variations in nitrogen accumulation (Figure 4). At both anthesis and maturity, the activities of glutamine synthetase (GS) and glutamate synthase (GOGAT) in the leaves of HLF were significantly higher than those of QV9 (*p* < 0.05), with higher activities occurring at anthesis. At the Guiyang site, the GS activity of HLF ranged from 12.17 to 15.68 U·g^−1^ FW at maturity, whereas that of QV9 was only 9.91–12.19 U·g^−1^ FW. Under BSN treatment, the GOGAT activity of HLF increased by 31.88% (Guiyang) and 155.63% (Anshun) compared to QV9. Furthermore, HLF exhibited significantly higher nitrate reductase (NR) activity compared with QV9 under both SN and BSN treatments. In contrast, NiR activity showed minimal variation across genotypes and nitrogen levels, and the only significant difference occurred between SN−HLF and BSN−QV9.

### 2.7. Antioxidant Enzyme Activities and Osmotic Adjustment Substances

Genotype and nitrogen application rate significantly affected the activities of protective enzymes (Figure 5). Compared with the SN treatment, BSN significantly increased the activities of superoxide dismutase (SOD), catalase (CAT), and peroxidase (POD), and HLF showed significantly higher SOD activity than QV9 under BSN treatment. At anthesis, HLF exhibited notably higher enzyme activities than QV9 at the Anshun site, with increases of 26.84%, 16.88%, and 3.36% for SOD, CAT, and POD, respectively.

Dynamic changes in osmotic adjustment substances are presented in Figure 6. Under BSN treatment, HLF accumulated significantly higher levels of proline (PRO) and soluble sugar content (SSC). At the Guiyang site, the PRO in HLF reached 407.60 μg·g^−1^ at maturity, significantly exceeding the 262.16 μg·g^−1^ recorded for QV9. Similarly, at the Anshun site, the SSC in HLF peaked at 7.71 mg·g^−1^, representing a 30.02% increase compared to QV9 (5.93 mg·g^−1^). Malondialdehyde (MDA), a key indicator of membrane lipid peroxidation, was significantly higher in QV9 than in HLF under BSN treatment at maturity (12.98% higher at Guiyang and 23.54% higher at Anshun). Collectively, these findings demonstrate that HLF effectively maintained cell turgor and metabolic function via osmotic adjustment under BSN treatment, thereby mitigating membrane damage and enhancing tolerance to nitrogen-deficient soils.

### 2.8. Correlation Analysis of Traits with NUE and Yield

As shown in Figure 7 and Table S1, Mantel tests revealed that nitrogen-use efficiency (NUE) was significantly correlated with DMAPP, TNA, NTR, GS, GOGAT, and POD (Mantel’s *p* < 0.05), and highly significantly correlated with DMTA, DMTR, DMTCR, GOGAT, SOD, SSC, PRO MDA, LAI and SPAD (Mantel’s *p* < 0.01). Similarly, yield showed highly significant correlations with TDMA, DMAP, GDMA, HI, DMAPP, DMTR, DMTCR, TNA, NAP, GNA, NHI, NAPP, NTA, NTR, NTCR, GS, GOGAT, NR, NiR, MDA, LAI and SPAD (Mantel’s *p* < 0.01). Notably, DMAPP (r = 0.167 and 0.412), TNA (r = 0.124 and 0.741), GS (r = 0.156 and 0.524), GOGAT (r = 0.361 and 0.388), MDA (r = 0.267 and 0.490), LAI (r = 0.394 and 0.382) and SPAD (r = 0.705 and 0.312) were commonly and significantly linked to both NUE and yield. Pearson correlation analysis among physiological traits showed that DMAPP was positively correlated with GDMA, GS, GOGAT, LAI, and SPAD, with correlation coefficients of 0.803, 0.872, 0.704, 0.700, and 0.788, respectively (*p* < 0.001). NAP was positively correlated with NR, GS, GOGAT, LAI, and SPAD, with r values of 0.615, 0.861, 0.650, 0.784, and 0.647, respectively (*p* < 0.001). MDA showed significant negative correlations with DMAPP, GS, GOGAT, LAI, and SPAD, with r values of −0.822, −0.794, −0.793, −0.705, and −0.856, respectively (*p* < 0.001) (Table S2).

## 3. Discussion

During the photosynthetic assimilation process, the activities of nitrogen metabolism enzymes are associated with nitrogen allocation; variation in the amide synthesis pathway may relate to differences in NUE [29,30]. Studies have consistently shown that key nitrogen metabolism enzymes, such as GS and GOGAT, exhibit higher activities in nitrogen-efficient genotypes, correlating with both nitrogen assimilation and remobilization [31,32]. In the present study, the nitrogen-efficient genotype HLF exhibited significantly higher activities of NR, GS, and GOGAT than the inefficient genotype QV9 across all nitrogen treatments (Figure 4). The GS-GOGAT cycle is the primary pathway for ammonium assimilation and is associated with the efficiency of converting inorganic nitrogen into organic forms [33]. Higher GS/GOGAT activities observed in HLF aligned with its superior yield, NUpE, and NUtE, all of which were significantly intercorrelated (Figure 7). In contrast, NiR activity showed limited variation across both genotypes and nitrogen levels, consistent with the view that NiR is conservatively regulated to prevent nitrite toxicity, whereas NR accounts for most of the genotypic and nitrogen-driven variation in assimilation capacity [34,35]. Accordingly, the elevated NR activity, together with higher GS and GOGAT activities, was likely associated with greater post-anthesis nitrogen accumulation (Pearson correlation coefficients between NAP and NR, GS, and GOGAT activities were 0.615 **, 0.861 **, and 0.650 **, respectively; Figure 7). However, direct measurements of nitrate and ammonium pools are needed to confirm this hypothesis.

Nitrogen, being a central component of chlorophyll, directly influences leaf chlorophyll content, which serves as a reliable proxy for nitrogen status [36]. Genotypes with high NUE typically exhibit a slower decline in SPAD values during grain filling compared to inefficient genotypes [37,38]. Consistent with this, HLF maintained higher SPAD values than QV9 under BSN treatment, particularly during the late reproductive stage (Figure 1). This chlorophyll retention complemented the enhanced GS and GOGAT enzyme activities discussed previously. Furthermore, HLF constructed a superior LAI trajectory: it achieved rapid early-stage growth and maintained consistently higher LAI values throughout the post-anthesis period (Figure 1). Given that the photosynthetic apparatus of C4 plants, such as sorghum, is highly sensitive to nitrogen allocation [39], the ability of HLF to maintain high LAI and chlorophyll stability under BSN treatment suggests that high NUE is associated not only with intrinsic metabolic traits but also with canopy architectural characteristics (Figure 7).

The accumulation and partitioning of photoassimilates are pivotal determinants of grain yield [40]. Our findings reveal that the two genotypes employed distinct source-sink regulatory strategies. Although QV9 exhibited higher dry matter and nitrogen translocation rates and their contributions to grains (Figure 2 and Figure 3), this pattern likely reflects a compensatory strategy, whereby the higher translocation rates in QV9 were associated with greater mobilization of stored reserves. In contrast, although HLF exhibited lower translocation rates under BSN, its proportions of post-anthesis dry matter and nitrogen accumulation (DMAPP and NAPP), dry matter and nitrogen accumulation of grain (GDMA and GNA) and nitrogen harvest index (NHI) were significantly higher than those of QV9 (Table 3 and Table 4). Notably, this finding diverges from the prevailing view in major cereals that nitrogen-efficient cultivars depend mainly on pre-anthesis assimilate translocation [41]. However, it is consistent with studies showing that genetic variations in grain nitrogen accumulation are more closely related to post-anthesis nitrogen uptake than to remobilization [42,43], suggesting a potentially distinctive nitrogen-use strategy in HLF that warrants validation across a broader range of genotypes and environments.

Under low nitrogen treatment, plants often face an increased risk of oxidative damage due to excess excitation energy at photosystem II, which can drive reactive oxygen species (ROS) generation and lipid peroxidation [44,45]. In the present study, QV9 exhibited higher malondialdehyde (MDA) accumulation under BSN treatment (Figure 6), indicating more severe membrane lipid peroxidation. By contrast, HLF displayed concurrent elevations in antioxidant enzyme activities (Figure 5) and accumulated compatible osmolytes such as proline (PRO) and soluble sugars (SSC) (Figure 6). These physiological traits were associated with enhanced oxidative stress tolerance and the preservation of osmotic balance under BSN treatment [46,47]. Such capacities aligned with its better performance compared with the N-inefficient genotype QV9.

A nutrient-efficient plant is defined as one that “produces higher economic yields from a given amount of nutrient input or uptake compared to a control or standard plant under identical growing conditions” [48]. In alignment with this definition, although the BSN treatment reduced grain yields in both genotypes, the yield reduction in HLF (approximately 11%) was substantially lower than that in QV9 (approximately 24–26%) (Table 1), confirming HLF as a nitrogen-efficient genotype. Regarding the components of NUE, Moll et al. [27] suggested that nitrogen uptake efficiency (NUpE) dominates under high soil nitrogen availability, whereas Ortiz-Monasterio et al. [49,50] emphasized that both NUpE and NUtE are critical under low-nitrogen conditions. Our data revealed that HLF’s superiority was most pronounced in NUpE and NUtE under BSN treatment (Table 2). This advantage coincided with its stronger post-anthesis biomass production (Figure 7; DMAPP showed significant positive correlations with both NUE and yield) and higher nitrogen metabolism enzyme activities (Figure 7; GS and GOGAT were significantly linked to both NUE and yield).

It is important to recognize that the identified associations may partly stem from mathematical dependencies among variables sharing computed components (e.g., yield in NUtE). Additionally, translocation metrics reflect apparent net changes rather than actual fluxes, omitting respiratory costs and unmeasured sinks. We also acknowledge that interpreting nitrogen uptake efficiency (NUpE) solely based on alkali-hydrolyzable nitrogen (AN) in the 0–20 cm topsoil has limitations: genotypic differences in root architecture and temporal patterns of nitrogen uptake may influence actual nitrogen acquisition efficiency beyond what is captured by AN measurements. Therefore, the NUpE estimates in this study should be regarded as relative indicators of genotypic nitrogen acquisition capacity rather than absolute values. Although such constraints are unavoidable in multi-site field trials, they do not detract from the physiological insights gained regarding HLF and QV9. After anthesis, HLF and QV9 diverged in their temporal trajectories: a progressive decline in LAI and SPAD, a reduction in NR and GS activities, and a concomitant accumulation of osmolytes. These changes reflect the sorghum developmental program. Future studies incorporating root phenotyping and continuous monitoring of soil nitrogen dynamics across soil profiles would provide a more comprehensive understanding of genotype-dependent nitrogen uptake strategies. Furthermore, integrating isotopic labeling, high-temporal resolution phenotyping, and structural equation modeling will help disentangle mathematical covariance from causal physiological networks and precisely quantify NUE-related translocation and senescence dynamics.

## 4. Materials and Methods

### 4.1. Experimental Site Description

Field experiments were conducted in 2024 at two experimental stations of the Guizhou Academy of Agricultural Sciences: Huaxi District, Guiyang (26°38′ N, 106°45′ E) and Xixiu District, Anshun (26°25′ N, 105°51′ E), Guizhou Province, China. Both sites are characterized by a subtropical humid monsoon climate. The soil at both locations was classified as clay loam with moderate fertility and uniform nutrient distribution. Prior to sowing, the physicochemical properties of the topsoil (0–20 cm) were determined. At the Guiyang site, the soil contained organic matter at 15.75 g·kg^−1^, alkali-hydrolyzable nitrogen at 95.71 mg·kg^−1^, available phosphorus at 21.49 mg·kg^−1^, available potassium at 114.62 mg·kg^−1^, had a pH of 7.60 and a bulk density of 1.21 g·cm^−3^. At the Anshun site, the corresponding values were 16.48 g·kg^−1^, 103.40 mg·kg^−1^, 25.86 mg·kg^−1^, 117.31 mg·kg^−1^, 7.65 and 1.11 g·cm^−3^, respectively.

### 4.2. Experimental Design

Two waxy sorghum genotypes with contrasting nitrogen-use efficiencies were used—a nitrogen-efficient cultivar (HLF) and a nitrogen-inefficient cultivar (QV9)—which were selected as extreme contrasting genotypes for nitrogen-use efficiency, identified through our previous two-year multi-location evaluation trials. Two nitrogen application rates were established: 0 kg N·ha^−1^ (BSN) and 150 kg N·ha^−1^ (SN). Phosphorus (P_2_O_5_) and potassium (K_2_O) were uniformly applied as base fertilizers at a rate of 75 kg·ha^−1^ across all treatments to avoid deficiency limitations, as soil available P and K were above the critical thresholds for sorghum production in both sites. In the BSN treatment, no nitrogen fertilizer was applied. In the SN treatment, 30% of the nitrogen was applied as basal dressing, while the remaining 70% was top-dressed at the jointing stage. The nitrogen, phosphorus, and potassium sources were coated urea (46% N), superphosphate (12% P_2_O_5_), and potassium sulfate (52% K_2_O), respectively.

The experimental field was deeply plowed in winter and 20 days before sowing. One day prior to sowing, rotary tillage was performed to finely pulverize the soil. The experiment was arranged in a randomized complete block design with three replicates at each site. Each plot consisted of 8 rows, 5 m in length, with a row spacing of 60 cm. Plants were thinned to two seedlings per hill, with 17 hills per row, resulting in a plot area of 24 m^2^. The plants were thinned at the five-leaf stage and manually weeded at the jointing stage. Sowing and harvesting dates varied slightly between locations: in Guiyang, sowing occurred on 18 April 2024, and harvesting on 24 August 2024; in Anshun, sowing was on 22 April 2024, and harvesting on 28 August 2024. Other field management practices were kept consistent across all plots and sites.

### 4.3. Measurements and Calculations

#### 4.3.1. Determination of Leaf Area Index (LAI) and Chlorophyll Content

At the jointing stage, anthesis stage, and 10, 20, 30 days after anthesis (DAA), five representative plants were sampled from each plot. The maximum length and width of each leaf of each plant were measured using a tape measure. The individual leaf area was calculated using the length–width coefficient method with a correction factor (K) of 0.723 [51]. The LAI was subsequently calculated based on the plant density per unit area. Simultaneously, the chlorophyll content of the functional leaves (the penultimate leaf) was measured using a portable chlorophyll meter (SPAD-502 Plus, Konica Minolta, Osaka, Japan). Five points were measured per leaf, and the average value was recorded.

#### 4.3.2. Determinations of Dry Matter and Nitrogen Accumulation and Translocation

At anthesis and maturity stage, five plants were harvested from each plot. The plants were partitioned into different organs: stem, leaf, sheath, panicle at anthesis; and stem, leaf, sheath, panicle, grain at maturity. Every organ was placed in an oven for 30 min at 105 °C to kill the fresh tissues and then dried to constant weight at 60 °C. After cooling to room temperature, the organs were weighed using an analytical balance (accuracy = 0.001 g). The dried samples were then ground to pass through a 60 mesh sieve, bagged, and labeled for analysis. The nitrogen content of each organ was determined using the Kjeldahl method (Kjeltec™ 8400, FOSS, Hillerød, Denmark). Nitrogen accumulation in each organ was calculated accordingly. Total dry matter accumulation (TDMA) and pre-anthesis dry matter accumulation (PDMA) are defined as the sum of dry matter weights of all organs per unit area at maturity and anthesis, respectively. Similarly, total nitrogen accumulation (TNA) and pre-anthesis nitrogen accumulation (PNA) are defined as the sum of nitrogen accumulation in all organs per unit area at maturity and anthesis, respectively.

Dry matter translocation amount (DMTA), dry matter translocation rate (DMTR), contribution rate of dry matter translocation to grains (DMTCR), post-anthesis dry matter accumulation (DMAP), proportion of post-anthesis dry matter accumulation (DMAPP), and harvest index (HI) in waxy sorghum were calculated using the following formulas described by Tong et al. [52] and Wang et al. [16]:

DMTA = PDMA − (TDMA − GDMA)(1)

DMTR = DMTA/PDMA × 100%(2)

DMTCR = DMTA/GDMA × 100%(3)

DMAP = TDMA − PDMA(4)

DMAPP = DMAP/TDMA × 100%(5)

HI = GDMA/TDMA × 100%(6)

where GDMA is dry matter accumulation of grain per unit area at maturity.

Nitrogen translocation amount (NTA), nitrogen translocation rate (NTR, %), contribution rate of nitrogen translocation to grain (NTCR), post-anthesis nitrogen accumulation (NAP) and proportion of post-anthesis nitrogen accumulation (NAPP), and nitrogen harvest index (NHI) in waxy sorghum were calculated using the following formulas described by Han et al. [53] and Wang et al. [16]:

NTA = PNA − (TNA − GNA)(7)

NTR = NTA/PNA × 100%(8)

NTCR = NTA/GNA × 100%(9)

NAP = TNA − PNA(10)

NAPP = NAP/TNA × 100%(11)

NHI = GNA/TNA × 100%(12)

where GNA is the N accumulation of grain per unit area at maturity.

#### 4.3.3. Measurements of Physiological Indices

At the anthesis stage, five uniform plants per plot were tagged for synchronized flowering monitoring. Samples were collected at anthesis (DAA0) and physiological maturity (DAA30). Leaf disks (2 cm diameter) were collected using a cork borer on the penultimate leaf at a rate of 10 disks per plant. The samples were immediately frozen in liquid nitrogen and stored at −80 °C until analysis. The activities of nitrate reductase (NR), nitrite reductase (NiR), glutamine synthetase (GS), and glutamate synthase (GOGAT) were assayed according to the methods described by Wang et al. [16]. The activities of superoxide dismutase (SOD), peroxidase (POD), and catalase (CAT) and malondialdehyde content (MDA) were assayed according to the methods described by GE et al. [54], and one unit of SOD activity was defined as the amount of enzyme required to cause 50% inhibition of NBT reduction, one unit of CAT activity was defined as the amount of enzyme that catalyzes the decomposition of 1 nmol of H_2_O_2_ caused by 1 g of leave sample per minute, one unit of POD activity was defined as the amount of enzyme that causes a change of 0.005 in absorbance at 470 nm caused by 1 g of leave sample per minute in a 1 mL reaction system. Soluble sugar content (SSC) was determined by the anthrone colorimetric method [55]. The proline content (PRO) was determined by the ninhydrin colorimetric method [56].

#### 4.3.4. Yield and Its Components

At the maturity stage, the central four rows of each plot were harvested, and the panicles were air-dried naturally. Ten representative panicles were selected for threshing to determine the weight and number of grains per panicle, the 1000-grain weight. The final grain yield was adjusted to a standard moisture content of 12.5% [57].

#### 4.3.5. Calculations of Nitrogen-Use Efficiency

The nitrogen uptake efficiency (NUpE), nitrogen utilization efficiency (NUtE), nitrogen partial factor productivity (NPFP), and nitrogen recovery efficiency (NRE) in waxy sorghum were calculated using the following formulas described by Moll et al. [27], Cao et al. [58] and Zheng et al. [59]:

Np = ANC × BD × SD × 10^−1^ (13)

NUpE = Nt/(Nr + Np)(14)

NUtE = Y/Nt(15)

NPFP= Y/Nr(16)

NRE = (Na − Nb)/Nr × 100%(17)

where ANC is alkali-hydrolyzable nitrogen content in the topsoil (0–20 cm), in mg·kg^−1^; BD is bulk density of topsoil (0–20 cm), in g·cm^−3^; SD is topsoil depth, set at 20 cm; 10^−1^ is the unit conversion factor; Np is the topsoil (0–20 cm) nitrogen supply capacity, in kg·ha^−1^; Nt is aboveground N accumulation; Nr is the current-season nitrogen application rate; Y is grain yield; Na and Nb are aboveground N accumulations in the SN treatment and the BSN treatment, respectively.

### 4.4. Statistical Analysis

Data were initially organized and plotted using Microsoft Excel 2010 (Microsoft Corp., Redmond, WA, USA). Analyses of variance (ANOVA) and mean comparisons were conducted separately for each site and, within each site, separately for each growth stage. A two-way ANOVA was performed using SPSS Statistics 20.0 (IBM Corp., Armonk, NY, USA) to assess the main effects of cultivar and nitrogen level and their interaction. Treatment means were compared using the least significant difference (LSD) test at *p* < 0.05. LSD post hoc comparisons were performed only within each corresponding site and growth stage analysis and were conducted following significant ANOVA results. Figures were finalized using Adobe Photoshop CS3 (Adobe Systems Inc., San Jose, CA, USA).

Additionally, to explore the linkage between physiological traits and agronomic performance, a Mantel test was conducted to assess the multivariate association between two Bray–Curtis distance matrices: (1) an agronomic performance matrix containing grain yield as an individual variable and nitrogen-use efficiency traits as a two-variable set (nitrogen uptake efficiency, NUpE; nitrogen utilization efficiency, NUtE); and (2) a physiological trait matrix containing all measured physiological parameters. Plot-level raw data (n = 24) from both sites (Guiyang and Anshun; 12 plots per site: 4 treatments × 3 replicates) were directly pooled into a single dataset with balanced site representation; both sites received identical experimental treatments and management. The TuTu Cloud Platform (https://cloudtutu.com/) was used, with 999 permutations for significance testing. No Z-score standardization was applied.

## 5. Conclusions

Under BSN treatment, the nitrogen-efficient waxy sorghum HLF maintained higher yield than QV9, which was associated with sustained post-anthesis accumulation, relatively elevated nitrogen metabolic enzyme activities, slower decline in canopy indicators (LAI and SPAD) and enhanced antioxidant enzyme activities, reflecting an integrated physiological response to low-nitrogen stress. HLF represents a promising genotype for improving NUE in waxy sorghum, and these physiological characteristics may support its potential for sustainable production in nitrogen-limited environments, pending multi-year multi-location validation.

## Figures and Tables

**Figure 1:**
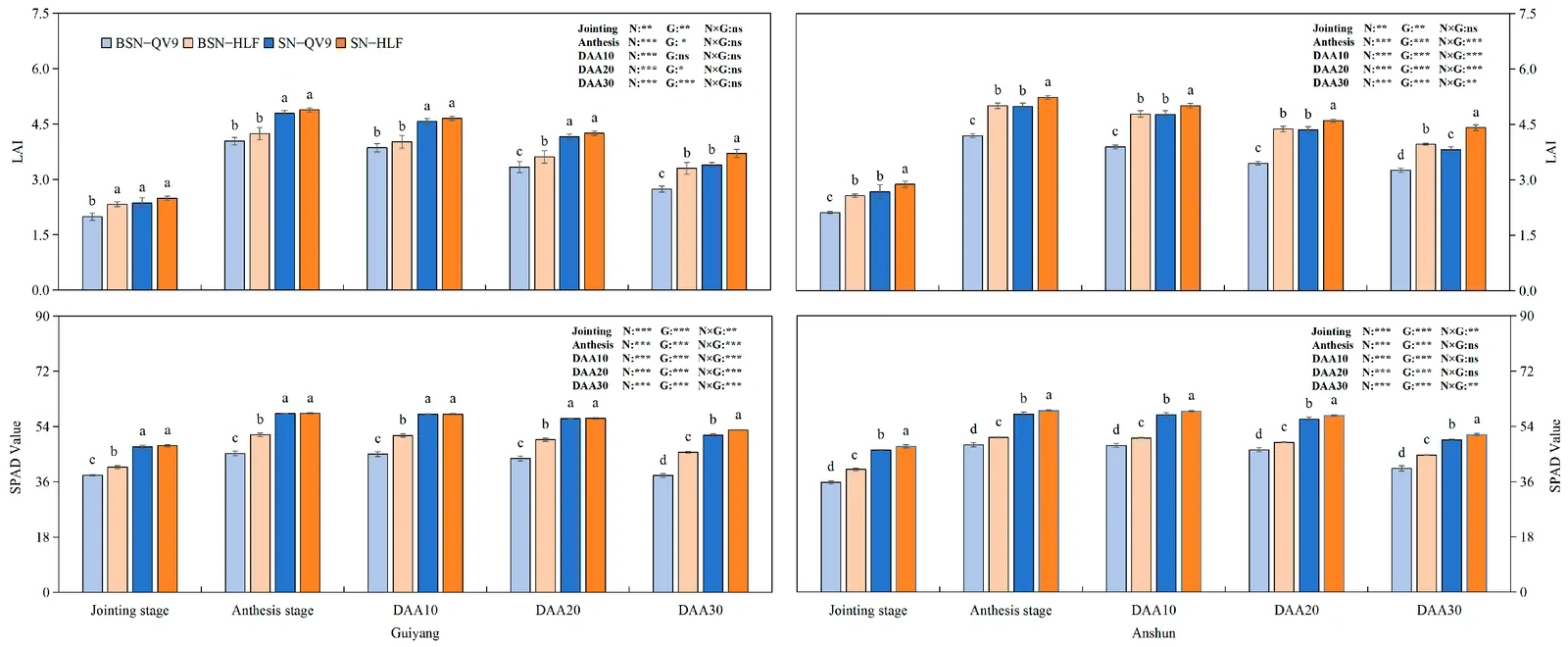
Leaf area index (LAI) and relative chlorophyll content (SPAD value) of waxy sorghum under different treatments. Data are the mean of three replicates and different lowercase letters indicate significant differences in LAI and SPAD value at the same stage and site among different treatments at the *p* = 0.05 level. ns, *, **, and *** indicate not significant and significant at the 0.05, 0.01, and 0.001 levels, respectively.

**Figure 2:**
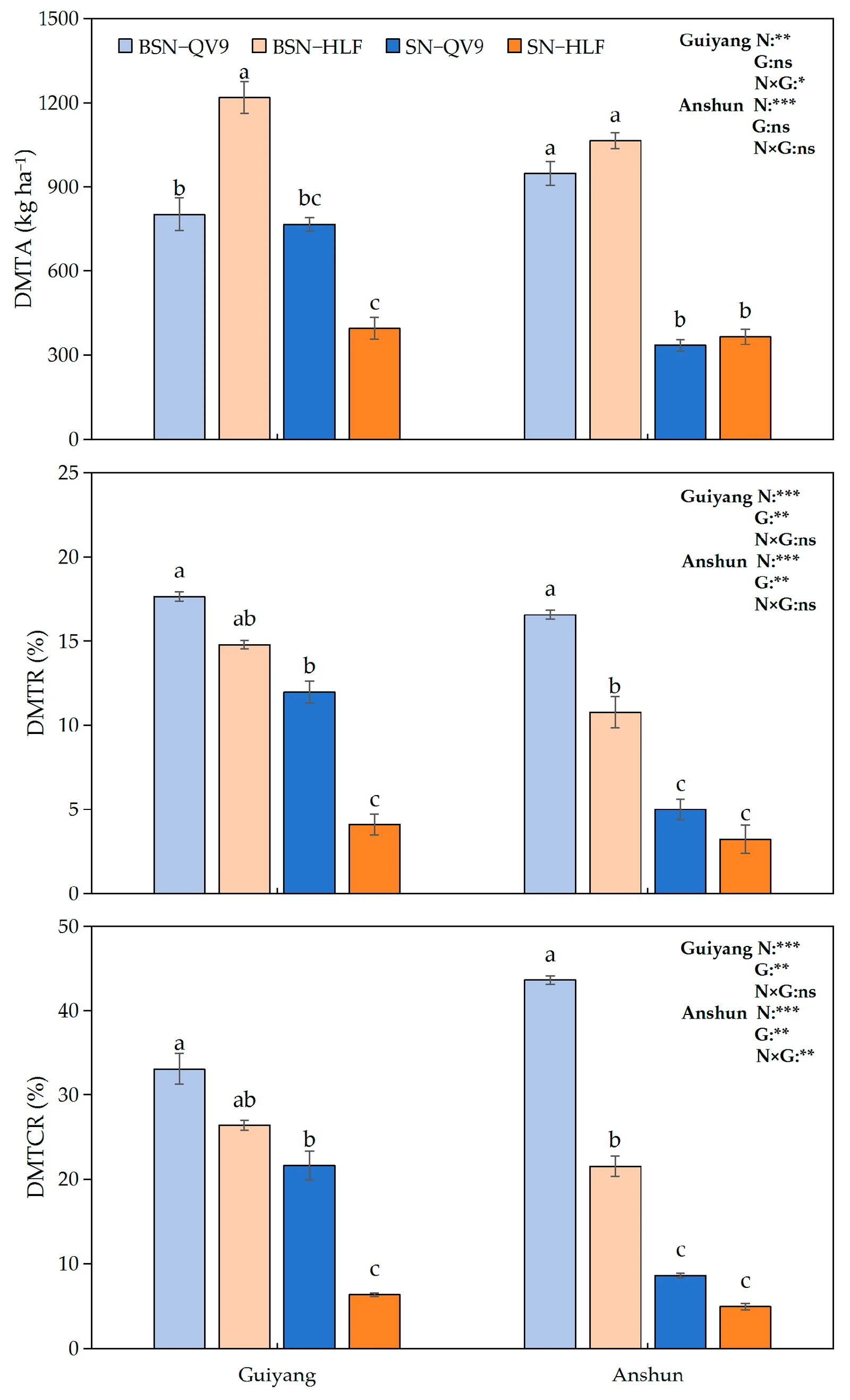
Dry matter translocation of waxy sorghum under different treatments. Data are the mean of three replicates and different lowercase letters indicate significant differences in DMTA, DMTR and DMTCR at the same site among different treatments at the *p* = 0.05 level. ns, *, **, and *** indicate not significant and significant at the 0.05, 0.01, and 0.001 levels, respectively.

**Figure 3:**
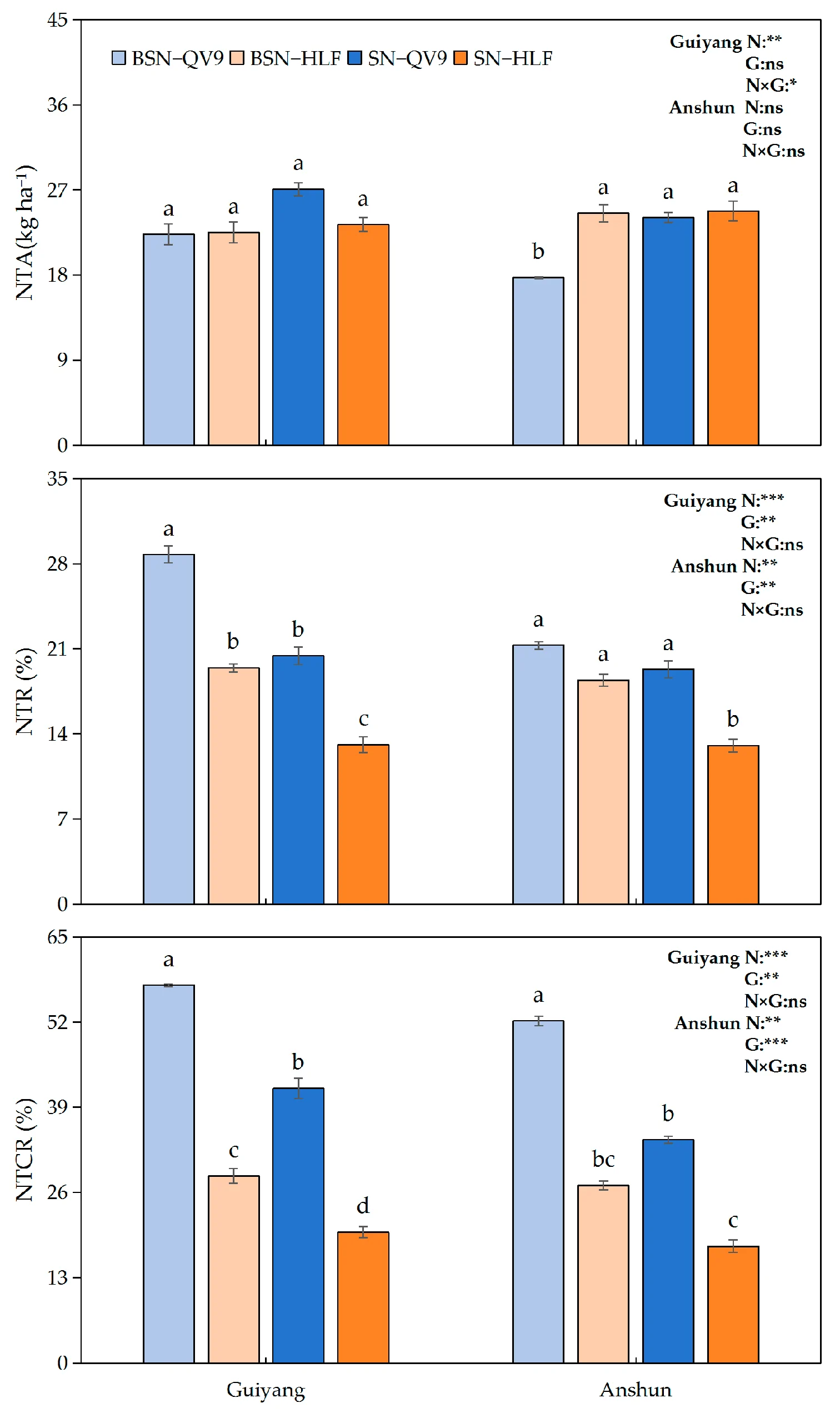
Nitrogen translocation of waxy sorghum under different treatments. Data are the mean of three replicates and different lowercase letters indicate significant differences in NTA, NTR and NTCR at the same site among different treatments at the *p* = 0.05 level. ns, *, **, and *** indicate not significant and significant at the 0.05, 0.01, and 0.001 levels, respectively.

**Figure 4:**
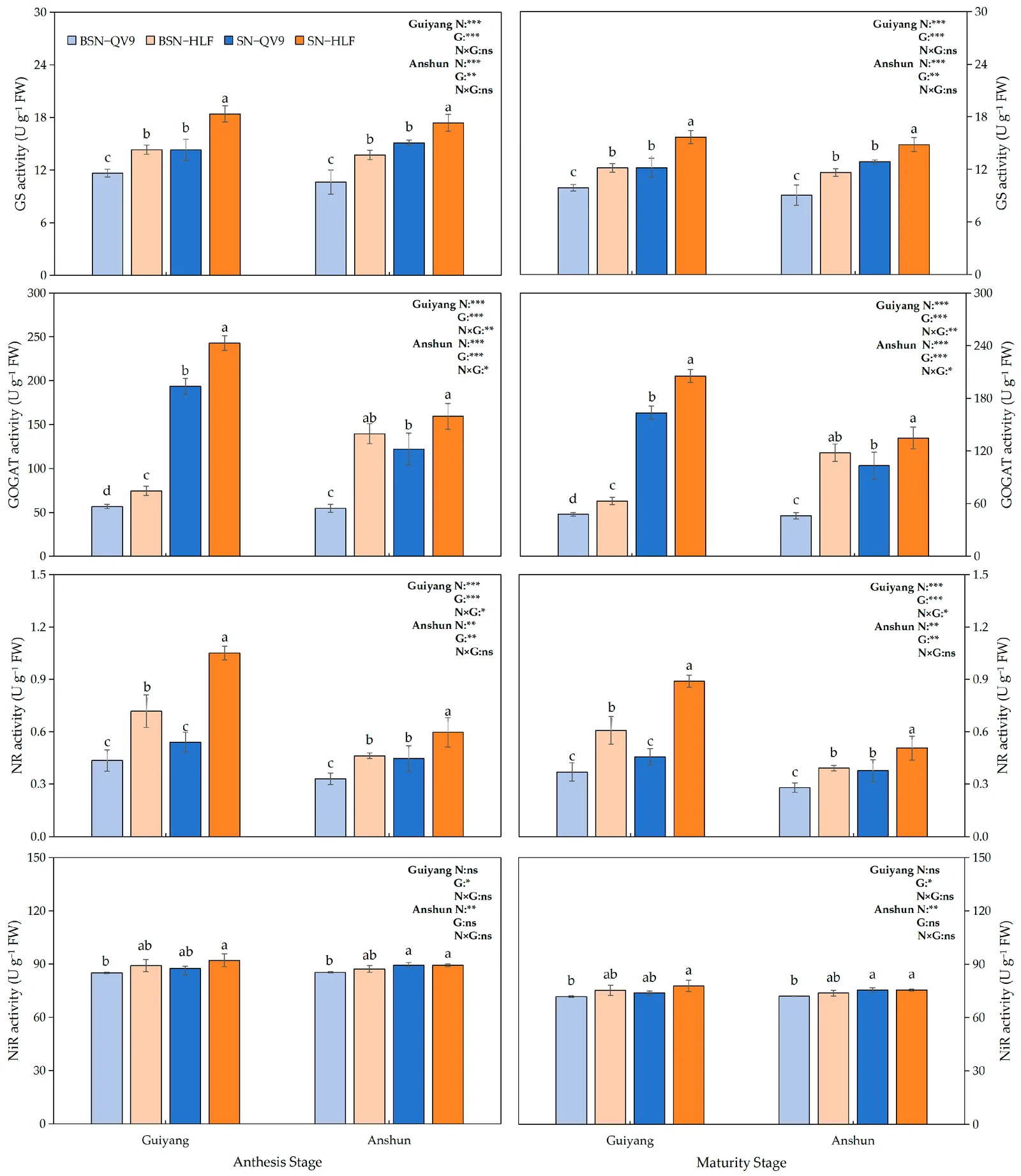
Changes in activities of nitrogen metabolism enzymes of waxy sorghum under different treatments. Data are the mean of three replicates and different lowercase letters indicate significant differences in GS, GOGAT, NR and NiR at the same stage and site among different treatments at the *p* = 0.05 level. ns, *, **, and *** indicate not significant and significant at the 0.05, 0.01, and 0.001 levels, respectively.

**Figure 5:**
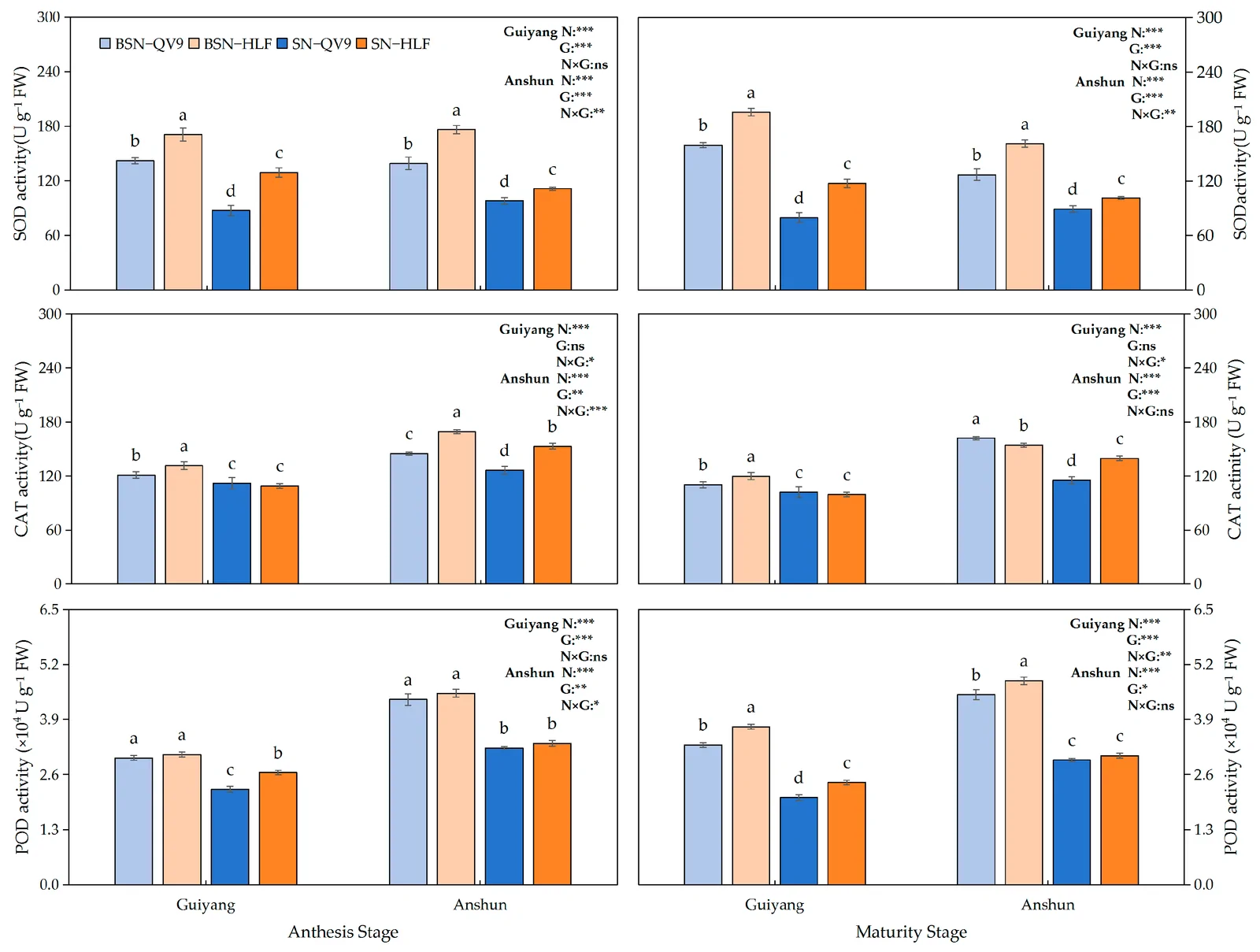
Changes in antioxidant enzyme activities of waxy sorghum under different treatments. Data are the mean of three replicates and different lowercase letters indicate significant differences in SOD, CAT and POD at the same stage and site among different treatments at the *p* = 0.05 level. ns, *, **, and *** indicate not significant and significant at the 0.05, 0.01, and 0.001 levels, respectively.

**Figure 6:**
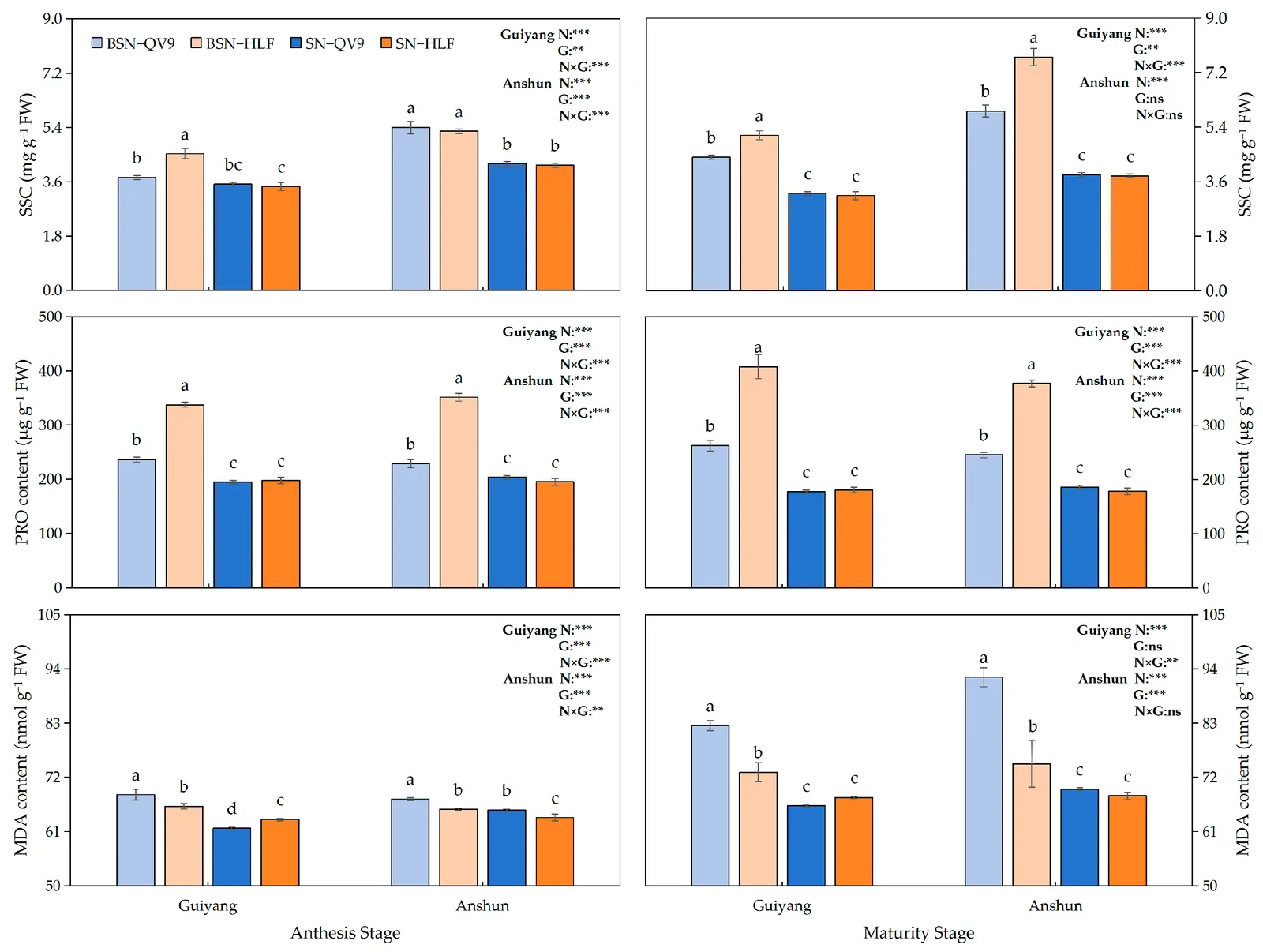
Changes in osmotic adjustment substances of waxy sorghum under different treatments. Data are the mean of three replicates and different lowercase letters indicate significant differences in SSC, PRO and MDA at the same stage and site among different treatments at the *p* = 0.05 level. ns, **, and *** indicate not significant and significant at the 0.01, and 0.001 levels, respectively.

**Figure 7:**
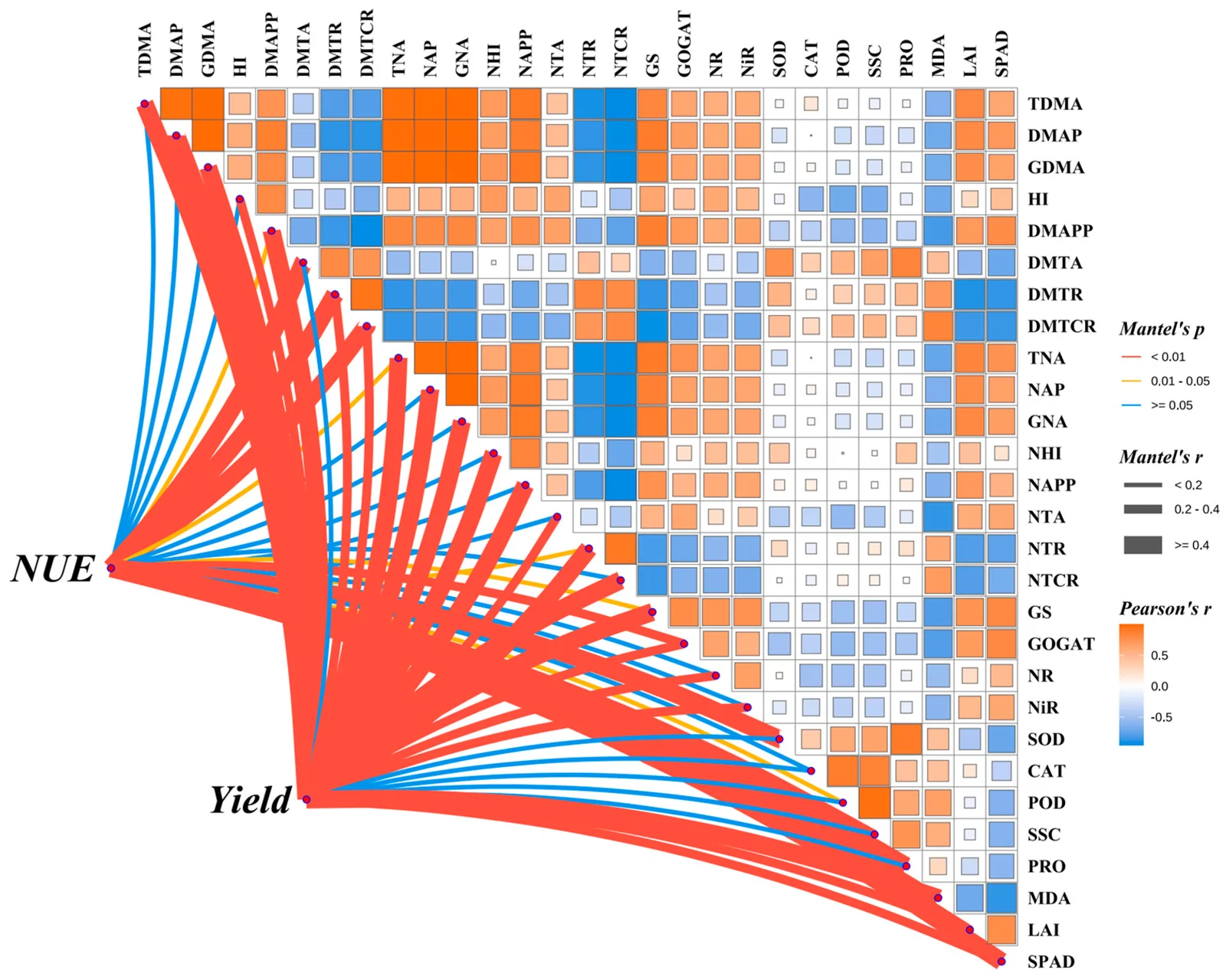
Heatmap and Mantel test of nitrogen-use efficiency (NUE), grain yield, and physiological traits of waxy sorghum. Enzyme activities and osmolytes were averaged at anthesis and maturity stage; SPAD and LAI were averaged across four stages (anthesis, DDA10, DDA20, DDA30). The upper triangle shows Pearson correlation coefficients (r) among the physiological traits, with color intensity indicating the direction and magnitude of the correlation (red: positive; blue: negative). The lower triangle presents the results of the Mantel test, comparing the Bray–Curtis distance matrix of these physiological traits with the NUE and yield distance matrix. Line width is proportional to the absolute value of Mantel’s r, and line color denotes significance levels (blue: *p* ≥ 0.05; orange: *p* < 0.05; red: *p* < 0.01).

**Table 1:** Yield and its components of waxy sorghum under different treatments.

Site	Treatment	Grain Yield (kg ha^−1^)	Yield Components of Waxy Sorghum		
			Grain Number per Spike	Grain Weight per Spike (g)	1000-Grain Weight (g)
Guiyang	BSN−QV9	2992.95 ± 9.56 d	1567.58 ± 53.24 c	27.14 ± 0.64 d	17.32 ± 0.34 d
	BSN−HLF	5861.34 ± 74.22 b	2711.36 ± 21.89 a	51.58 ± 0.51 b	19.02 ± 0.06 c
	SN−QV9	3954.15 ± 36.51 c	1746.86 ± 15.84 b	34.89 ± 0.32 c	19.97 ± 0.16 b
	SN−HLF	6588.68 ± 109.68 a	2667.12 ± 151.90 a	55.88 ± 0.70 a	20.95 ± 0.10 a
	Source of variation				
	N	***	ns	***	***
	G	***	***	***	***
	N × G	*	*	**	*
Anshun	BSN−QV9	2757.23 ± 58.72 d	1559.20 ± 32.05 d	26.83 ± 0.17 d	17.21 ± 0.28 d
	BSN−HLF	6094.13 ± 65.81 b	2806.77 ± 56.30 b	53.19 ± 0.19 b	18.95 ± 0.07 c
	SN−QV9	3741.63 ± 36.97 c	1697.74 ± 21.71 c	33.02 ± 0.32 c	19.45 ± 0.10 b
	SN−HLF	6807.76 ± 74.35 a	2893.90 ± 39.77 a	59.93 ± 0.64 a	20.71 ± 0.26 a
	Source of variation				
	N	***	**	***	***
	G	***	***	***	***
	N × G	**	ns	*	ns

Data are the mean of three replicates and different lowercase letters within a column at the same site indicate significant differences among treatments at the 0.05 level. BSN: 0 kg N·ha^−1^; SN: 150 kg N·ha^−1^; N: nitrogen application rate; G: genotype; N ×G: interaction between nitrogen application rate and genotype. ns, *, **, and *** indicate not significant and significant at the 0.05, 0.01, and 0.001 levels, respectively.

**Table 2:** Nitrogen-use efficiency of waxy sorghum under different treatments.

Site	Treatment	NUpE (kg kg^−1^)	NUtE (kg kg^−1^)	NPFP (kg kg^−1^)	NRE (%)
Guiyang	BSN−QV9	0.41 ± 0.03 c	31.81 ± 0.10 b	NA	NA
	BSN−HLF	0.74 ± 0.04 a	34.13 ± 0.43 a	NA	NA
	SN−QV9	0.45 ± 0.01 b	23.26 ± 0.21 d	26.36 ± 0.24 b	50.62 ± 3.52 b
	SN−HLF	0.71 ± 0.03 a	24.29 ± 0.40 c	43.92 ± 0.73 a	66.36 ± 5.63 a
	Source of variation				
	N	ns	***	NA	NA
	G	***	***	***	*
	N × G	**	**	NA	NA
Anshun	BSN−QV9	0.44 ± 0.02 c	27.54 ± 0.59 b	NA	NA
	BSN−HLF	0.87 ± 0.03 a	30.61 ± 0.33 a	NA	NA
	SN−QV9	0.45 ± 0.01 c	21.85 ± 0.22 c	24.94 ± 0.24 b	47.43 ± 1.86 b
	SN−HLF	0.80 ± 0.06 b	22.43 ± 0.24 c	45.39 ± 0.49 a	69.65 ± 8.38 a
	Source of variation				
	N	*	***	NA	NA
	G	***	***	***	*
	N × G	**	***	NA	NA

Data are the mean of three replicates and different lowercase letters within a column at the same site indicate significant differences among treatments at the 0.05 level. BSN: 0 kg N·ha^−1^; SN: 150 kg N·ha^−1^; N: nitrogen application rate; G: sorghum genotype; N × G: interaction between nitrogen application rate and sorghum genotype. NA indicates not applicable. *, **, and *** indicate significance at the 0.05, 0.01, and 0.001 levels, respectively.

**Table 3:** Dry matter accumulation and partitioning of waxy sorghum under different treatments.

Site	Treatment	TDMA (kg ha^−1^)	DMAP (kg ha^−1^)	GDMA (kg ha^−1^)	HI (%)	DMAPP (%)
Guiyang	BSN−QV9	6180.04 ± 319.45 d	1622.12 ± 53.95 d	2424.21 ± 73.60 d	39.27 ± 1.57 a	26.26 ± 0.48 c
	BSN−HLF	11,559.12 ± 348.57 b	3400.21 ± 307.26 b	4617.98 ± 45.05 b	39.97 ± 0.97 a	29.48 ± 3.42 b
	SN−QV9	9169.85 ± 67.60 c	2787.64 ± 229.92 c	3554.34 ± 68.13 c	38.76 ± 0.48 a	30.39 ± 2.28 b
	SN−HLF	15,377.16 ± 113.65 a	5815.55 ± 272.40 a	6211.27 ± 95.75 a	40.39 ± 0.34 a	37.82 ± 1.66 a
	Source of variation					
	N	***	***	***	ns	**
	G	***	***	***	ns	**
	N × G	*	**	**	ns	ns
Anshun	BSN−QV9	6958.33 ± 173.16 d	1239.72 ± 19.68 c	2187.63 ± 62.56 d	31.41 ± 1.89 c	17.77 ± 0.42 c
	BSN−HLF	13,720.28 ± 165.48 b	3874.89 ± 22.92 b	4939.32 ± 73.64 b	35.99 ± 1.37 b	28.24 ± 0.46 b
	SN−QV9	10,205.60 ± 54.73 c	3539.52 ± 94.04 b	3874.20 ± 30.15 c	37.96 ± 0.09 b	34.68 ± 0.98 a
	SN−HLF	18,331.37 ± 235.60 a	7020.51 ± 50.50 a	7386.51 ± 77.61 a	40.29 ± 0.45 a	38.29 ± 0.96 a
	Source of variation					
	N	***	***	***	***	***
	G	***	***	***	**	***
	N × G	**	*	*	ns	*

Data are the mean of three replicates and different lowercase letters within a column at the same site indicate significant differences among treatments at the 0.05 level. BSN: 0 kg N·ha^−1^; SN: 150 kg N·ha^−1^; N: nitrogen application rate; G: sorghum genotype; N × G: interaction between nitrogen application rate and sorghum genotype. ns, *, **, and *** indicate not significant and significant at the 0.05, 0.01, and 0.001 levels, respectively.

**Table 4:** Nitrogen accumulation and partitioning of waxy sorghum under different treatments.

Site	Treatment	TNA (kg ha^−1^)	NAP (kg ha^−1^)	GNA (kg ha^−1^)	NHI (%)	NAPP (%)
Guiyang	BSN−QV9	94.09 ± 4.85 c	16.38 ± 1.16 d	38.68 ± 0.89 d	41.16 ± 1.63 b	17.42 ± 0.98 c
	BSN−HLF	171.73 ± 5.95 b	56.45 ± 3.54 b	78.97 ± 0.92 b	46.02 ± 1.27 a	32.94 ± 3.17 a
	SN−QV9	170.03 ± 0.50 b	37.62 ± 3.29 c	64.71 ± 0.71 c	38.06 ± 0.35 c	22.13 ± 1.93 b
	SN−HLF	271.27 ± 4.94 a	93.76 ± 5.22 a	117.10 ± 3.33 a	43.16 ± 0.55 b	34.56 ± 1.61 a
		Source of variation				
	N	***	***	***	**	*
	G	***	***	***	***	***
	N × G	**	**	***	ns	ns
Anshun	BSN−QV9	100.11 ± 3.22 d	16.98 ± 4.73 d	34.31 ± 2.99 d	34.23 ± 1.86 c	16.88 ± 1.27 c
	BSN−HLF	199.07 ± 4.32 b	66.08 ± 4.54 b	90.62 ± 5.14 b	45.50 ± 1.65 a	33.19 ± 1.98 a
	SN−QV9	171.26 ± 0.49 c	46.67 ± 1.98 c	70.74 ± 0.39 c	41.31 ± 0.31 b	27.25 ± 1.08 b
	SN−HLF	303.55 ± 8.40 a	114.14 ± 4.93 a	138.91 ± 6.10 a	45.75 ± 1.06 a	37.61 ± 1.42 a
		Source of variation				
	N	***	***	***	**	**
	G	***	***	***	***	***
	N × G	***	*	*	**	ns

Data are the mean of three replicates and different lowercase letters within a column at the same site indicate significant differences among treatments at the 0.05 level. BSN: 0 kg N·ha^−1^; SN: 150 kg N·ha^−1^; N: nitrogen application rate; G: sorghum genotype; N × G: interaction between nitrogen application rate and sorghum genotype. ns, *, **, and *** indicate not significant and significant at the 0.05, 0.01, and 0.001 levels, respectively.

## Data Availability

The original contributions presented in this study are included in the article and Supplementary Materials. The Supplementary Materials provide: (1) raw data tables for all measured parameters (used to generate Figures and Tables); (2) the plot-level raw matrices for Mantel tests (n = 24, including physiological traits and agronomic performance under both sites); and (3) information on the experimental germplasm sources. Further inquiries can be directed to the corresponding author.

## Supplementary Materials

The following supporting information can be downloaded at: https://www.mdpi.com/article/10.3390/plants15193037/s1, Table S1. Mantel’s r and p-values between physiological traits and nitrogen-use efficiency (NUE) or grain yield in waxy sorghum. Table S2. Pearson correlation coefficients among physiological traits of waxy sorghum.
