# Supplementary material for: Physiological Determinants of High Nitrogen-Use Efficiency in Waxy Sorghum: Integrated Analysis of Biomass Production, Nitrogen Assimilation, and Antioxidant Metabolism
Source: Plants (Basel). 2026 Oct 4;15(19):3037. doi: 10.3390/plants15193037 (PMC13666668; doi:10.3390/plants15193037)
Supplement: Supplementary file 1 [file plants-15-03037-s001.zip › plants-4564093-supplementary.pdf]

Table S1. Mantel's r and p-values between physiological traits and nitrogen-use efficiency (NUE) or grain yield in waxy sorghum.

Trait	NUE		Yield	
	r	p-value	r	p-value
TDMA	0.091	0.076	0.862	0.001
DMAP	0.070	0.115	0.739	0.001
GDMA	0.084	0.086	0.877	0.001
HI	-0.053	0.745	0.304	0.001
DMAPP	0.167	0.017	0.412	0.001
DMTA	0.602	0.001	0.088	0.087
DMTR	0.427	0.001	0.241	0.009
DMTCR	0.428	0.001	0.332	0.002
TNA	0.124	0.041	0.741	0.001
NAP	0.080	0.095	0.843	0.001
GNA	0.085	0.081	0.840	0.001
NHI	-0.013	0.518	0.584	0.001
NAPP	-0.002	0.417	0.828	0.001
NTA	0.009	0.354	0.317	0.001
NTR	0.138	0.025	0.503	0.001
NTCR	0.094	0.069	0.848	0.001
GS	0.156	0.015	0.524	0.001
GOGAT	0.361	0.002	0.388	0.001
NR	-0.035	0.730	0.360	0.001
NiR	0.106	0.061	0.249	0.004
SOD	0.795	0.001	0.046	0.167
CAT	-0.011	0.472	0.034	0.204
POD	0.124	0.039	0.007	0.404
SSC	0.259	0.005	0.015	0.344
PRO	0.748	0.001	0.104	0.067
MDA	0.267	0.005	0.490	0.001
LAI	0.394	0.002	0.383	0.003
SPAD	0.705	0.001	0.312	0.004

Agronomic performance matrix containing grain yield (Yield) as an individual variable and nitrogen-use efficiency traits as a two-variable set (nitrogen uptake efficiency, NUpE; nitrogen utilization efficiency, NUtE). Enzyme activities and osmolytes were averaged at anthesis and maturity stage; SPAD and LAI were averaged across four stages (anthesis, DDA10, DDA20, DDA30).

Table S2. Pearson correlation coefficients among physiological traits of waxy sorghum.

Trait	DMAP	GDMA	DMAPP	NAP	NAPP	GS	GOGAT	NR	MDA	LAI	SPAD
DMAP	1										
GDMA	0.985**	1									
DMAPP	0.864**	0.803**	1								
NAP	0.989**	0.994**	0.804**	1							
NAPP	0.891**	0.920**	0.771**	0.921**	1						
GS	0.893**	0.863**	0.872**	0.861**	0.757**	1					
GOGAT	0.681**	0.642**	0.704**	0.650**	0.516**	0.785**	1				
NR	0.603**	0.618**	0.594**	0.615**	0.578**	0.731**	0.635**	1			
MDA	-0.686**	-0.673**	-0.822**	-0.643**	-0.619**	-0.794**	-0.793**	-0.531**	1		
LAI	0.803**	0.778**	0.700**	0.784**	0.701**	0.737**	0.685**	0.238	-0.705**	1	
SPAD	0.707**	0.642**	0.788**	0.647**	0.533**	0.814**	0.814**	0.486*	-0.856**	0.777**	1

Pearson correlation coefficients are showed in the lower triangle. * and ** indicate significance at the 0.05 and 0.01 levels (two-tailed), respectively.
